# Unraveling the Binding Mechanism of Alzheimer’s Drugs with Irisin: Spectroscopic, Calorimetric, and Computational Approaches

**DOI:** 10.3390/ijms23115965

**Published:** 2022-05-25

**Authors:** Rashid Waseem, Anas Shamsi, Tanzeel Khan, Md. Imtaiyaz Hassan, Syed Naqui Kazim, Mohammad Shahid, Asimul Islam

**Affiliations:** 1 Centre for Interdisciplinary Research in Basic Sciences, Jamia Millia Islamia, New Delhi 110025, India; rashid.waseem439@gmail.com (R.W.); anas.shamsi18@gmail.com (A.S.); aligbcb11@gmail.com (T.K.); mihassan@jmi.ac.in (M.I.H.); skazim@jmi.ac.in (S.N.K.); 2 Department of Basic Medical Sciences, College of Medicine, Prince Sattam Bin Abdulaziz University, Al-Kharj 11942, Saudi Arabia; dr.shahid90@yahoo.com

**Keywords:** irisin, molecular docking, fluorescence spectroscopy, drug delivery, Alzheimer’s disease, isothermal titration calorimetry

## Abstract

The prevalence of Alzheimer’s disease (AD) has been a major health concern for a long time. Despite recent progress, there is still a strong need to develop effective disease-modifying therapies. Several drugs have already been approved to retard the progression of AD-related symptoms; however, there is a need to develop an effective carrier system for the delivery of drugs to combat such diseases. In recent years, various biological macromolecules, including proteins, have been used as carriers for drug delivery. Irisin is a beneficial hormone in such diseases, including AD and related pathologies. Herein, the interaction mechanism of irisin with AD drugs such as memantine, galantamine, and fluoxetine is investigated. Fluorescence studies revealed that the above drugs bind to irisin with significant affinity, with fluoxetine having the highest binding affinity. Isothermal titration calorimetry (ITC) complemented the spontaneous binding of these drugs with irisin, delineating various associated thermodynamic and binding parameters. Molecular docking further validated the fluorescence and ITC results and unfolded the mechanism that hydrogen bonding governs the binding of fluoxetine to irisin with a significant binding score, i.e., −6.3 kcal/mol. We believe that these findings provide a promising solution to fight against AD as well as a platform for further research to utilize irisin in the drug-delivery system for an effective therapeutic strategy.

## 1. Introduction

Alzheimer’s disease (AD) is the most prevalent neurological disease worldwide, pathologically characterized by late-stage amyloid β (Aβ) plaques, neurofibrillary tangles (NFTs), and neuronal cell death [1]. Around 50–55 million people worldwide are known to be affected by dementia, including AD, and it is predicted that its prevalence may reach up to 150 million by 2050 [2]. Many theories about the causes of AD exist, but the reason underlying its pathogenesis has not been understood completely; hence, there has been a hindrance in the development of new therapeutics for the cure of the AD. The accumulated Aβ plaques and NFTs caused by abnormally hyperphosphorylated tau protein are the pathological hallmarks of AD. The accumulation of Aβ plaques forms amyloid senile plaques (SPs), which, along with NFTs, have been intricately involved in the pathogenesis of AD [3]. It is believed that when Aβ peptides are produced excessively, they accumulate to form senile plaques resulting in neuronal death [3]. Over the last few years, AD treatment has mainly involved Aβ-targeting therapies, and various drug candidates targeting Aβ are also in clinical trials [2]. Along with Aβ, the tau pathway also has an important role in AD pathology and is receiving much attention for potential AD therapeutics [4].

Since AD has a multifactorial origin, current therapeutic approaches have not successfully addressed the root cause of its pathogenesis and are used for symptomatic treatment [5,6]. Although many drug candidates targeting Aβ and tau pathology are in the clinical phase, they provide only limited symptomatic relief. None can effectively slow down or halt the disease’s progression. Most of the drugs that have been approved for symptomatic treatment of mild to moderate dementia in AD and associated pathologies work mainly on the N-Methyl-D-Aspartate (NMDA) receptor or the cholinergic pathway [7]. Since synaptic dysfunction and memory impairment are common features of AD pathogenesis, the restoration of synapse function and memory is important in AD management.

Developing an effective carrier system for drug delivery is essential to combat various diseases. Many drug carriers, including magnetic nanoparticles, hydrogels, liposomes, and microspheres, have been developed for the treatment and diagnosis of various diseases [8,9]. However, finding an appropriate drug carrier system is not an easy task. Drug carrier systems have several shortcomings; poor stability, the undesired release of drugs, low drug-loading content, high cost, and toxicity. A good drug carrier system should have specific properties such as non-immunogenicity, optimal bioavailability, the ability to target a specific site, and non-toxic behavior. Natural biological molecules such as proteins and polysaccharides have excellent drug carrier properties. In recent research, the paradigm of the drug carrier system has been shifting to the use of biological macromolecules to deliver therapeutic drugs. Many investigators use proteins such as silk fibroin, transferrin, and albumin for drug carrier systems [10,11,12]. Irisin is a newly identified hormone known to have a protective effect against synapse failure and cognitive decline in AD-related pathologies [13]. It has been reported that irisin is secreted in the heart, liver, pancreas, and testes, and it crosses the blood–brain barrier [13,14].In the brain, irisin regulates brain-derived neurotrophic factor (BDNF) expression and helps in protecting the neuronal damage caused by oxidative stress [15,16].

**Figure 1:**
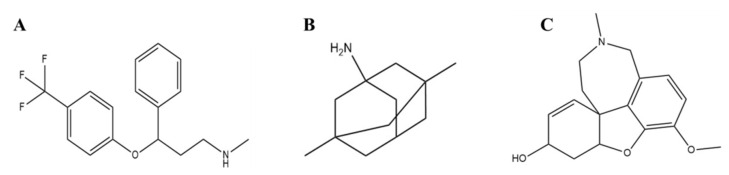
Two-dimensional structures of (**A**) Fluoxetine, (**B**) Memantine and (**C**) Galantamine.

Moreover, it has also been demonstrated that FNDC5/irisin interacts with the amyloid precursor protein (APP) and regulates Aβ level in the brain [14,17]. Therefore, it is speculated that using irisin as a drug carrier for the drugs approved for AD-related pathology could be a promising strategy for treating AD. Memantine, galantamine, rivastigmine, etc., are some approved drugs for treating mild to moderate AD symptoms. Figure 1 shows the structure of all these drugs. Memantine is a non-competitive NMDA receptor antagonist, which protects neurons against the overstimulation of NMDA receptors in the case of AD [18,19]. Galantamine is a cholinergic drug that inhibits the acetylcholinesterase (AChE) enzyme and alters the nicotinic cholinergic receptors, thereby counteracting AD [20,21]. Fluoxetine is another FDA-approved drug, which is a selective serotonin reuptake inhibitor (SSRI) antidepressant. Fluoxetine exerts its action through inhibition of serotonin uptake by nerve cells (neurons) and helps people with anxiety, depression, or obsessive-compulsive symptoms [22,23]. Herein, we have studied the interaction between irisin and AD drugs (memantine, galantamine, and fluoxetine) implicated in treating AD-related pathology. We have employed fluorescence spectroscopy, ITC, and molecular docking to elucidate the interaction pattern of irisin with these drugs and to decipher the binding mechanism.

## 2. Results and Discussion

### 2.1. Fluorescence Spectroscopic Measurements

Fluorescence spectroscopy is a commonly deployed technique for studying protein–ligand interaction, as it reveals various binding parameters as well as an understanding of the strength of the interaction [24]. Intrinsic fluorescence reveals information about the local microenvironment of aromatic amino acids, aiding in the investigation of the formation of the protein–ligand complex [25,26]. A decline in the fluorescence intensity of proteins with increasing ligand concentrations is termed fluorescence quenching [27].

**Figure 2:**
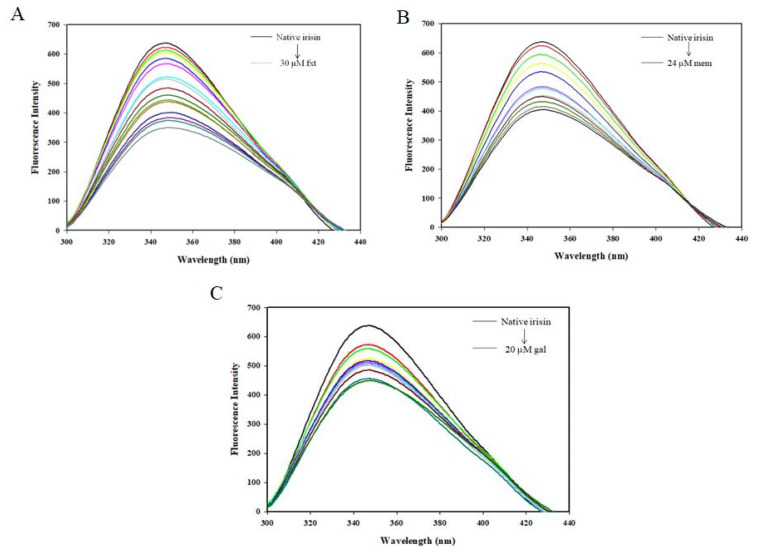
The fluorescence spectra of irisin in the presence of (**A**) Fluoxetine (0–30 μM), (**B**) Memantine (0–24 μM), and (**C**) Galantamine (0–20 μM).

Herein, the fluorescence quenching was observed for irisin–fluoxetine, irisin–memantine, and irisin–galantamine interactions. Fluorescence emission spectra of free irisin and irisin with different concentrations of fluoxetine (0–30 µM), memantine (0–24 µM), and galantamine (0–20 µM) are shown in Figure 2. The native irisin shows fluorescence emission maxima around 344 nm [28]. The fluorescence quenching of irisin was observed with increasing ligand concentrations for all three drugs. Fluorescence quenching was mathematically analyzed by applying (1) the Stern–Volmer (SV) and (2) double logarithmic equations to determine various quenching and binding parameters of protein–drug complexes as per previous reports [24,29].

(1) $$\frac{F_{0}}{F}=1+K_{sv}\left[C\right]$$

(2) $$log\frac{F_{0}-F}{F}=logK+nlog\left[C\right]$$

where *F*_0_ refers to the intensity of free irisin and *F* is the intensity of irisin in the presence of the drug. [*C*] denotes the varying concentration of drugs. *K_sv_* is the resulting Stern–Volmer constant.

*K* refers to the “binding constant of the irisin-drug complex”, *C* is the drug concentration, and “*n*” denotes the number of binding sites.

**Figure 3:**
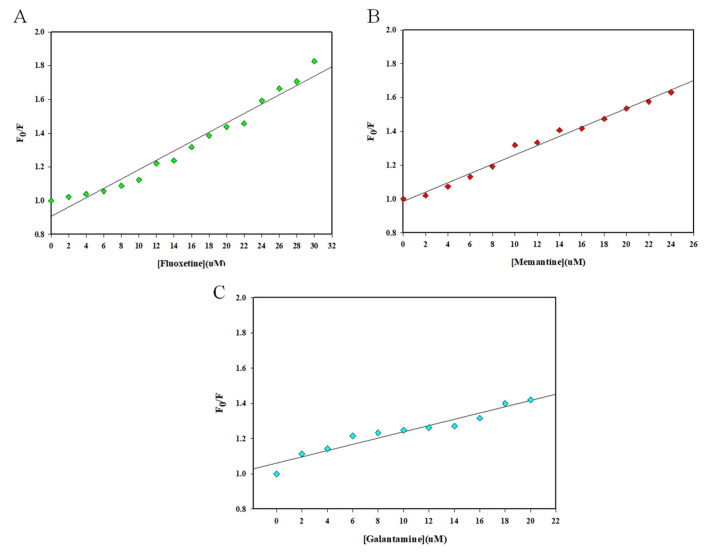
Stern–Volmer plot for the quenching of irisin fluorescence by (**A**) Fluoxetine, (**B**) Memantine, and (**C**) Galantamine.

**Figure 4:**
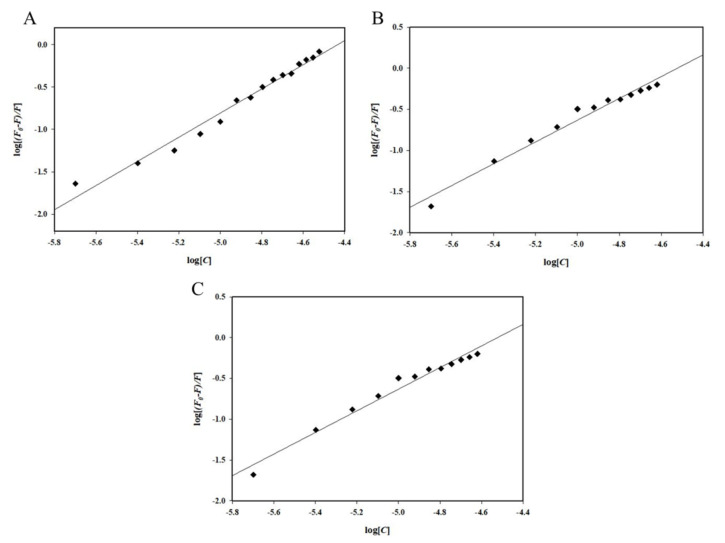
Double logarithmic plot for the quenching of irisin fluorescence by (**A**) Fluoxetine, (**B**) Memantine, and (**C**) Galantamine.

Figure 3 depicts SV plots of *F_0_/F* on the *y*-axis and [Fluoxetine], [Memantine], and [Galantamine] on the *x*-axis. The slope of the SV plot gives the Stern–Volmer constant (*K_sv_*) value using the SV equation (Equation (1)), which was highest for the irisin–fluoxetine interaction, i.e., 2.77 × 10^4^ M^−1^. The values of the *K_sv_* are listed in Table 1. To estimate the binding constants and number of binding sites, a double logarithmic plot (Equation (2)) was used [24]. The intercept of this plot provides the binding constant (*K*), and the slope of this plot gives the number of binding sites (*n*). Figure 4 depicts the double logarithmic plot for all three drugs, while Table 2 lists the binding parameters obtained. It was observed that fluoxetine binds to irisin with the highest affinity (*K* = 0.21 × 10^7^M^−1^ at 25 °C). Memantine and galantamine also showed significant binding to irisin, with a binding constant (*K*) of 9.78 × 10^5^M^−1^ and 0.14 × 10^3^M^−1^, respectively. Thus, it can be inferred that each of these AD drugs binds to irisin, forming a stable complex, with fluoxetine having the maximum affinity among the three drugs.

### 2.2. Isothermal Titration Calorimetry (ITC)

**Figure 5:**
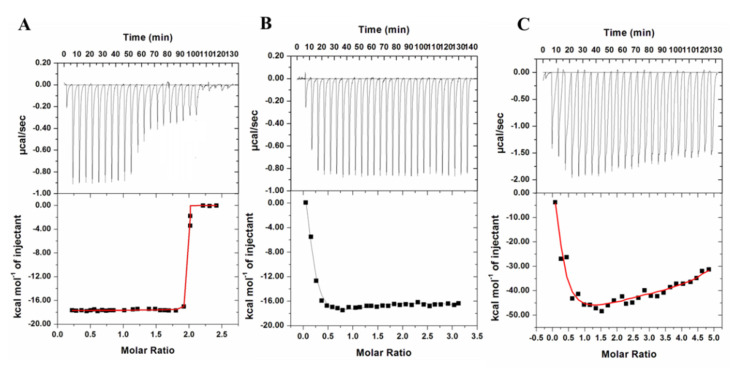
ITC profile of (**A**) Irisin–Fluoxetine, (**B**) Irisin–Memantine, and (**C**) Irisin–Galantamine systems. The top panels show raw data obtained upon sequentially titrating ligand into the protein of interest (irisin) present in the sample cell. The bottom panels show the binding isotherm obtained upon plotting the calorimetric titration’s integrated heat results after the correction of dilution heat against the molar ratio.

A complete description of the binding energetic was delineated by isothermal titration calorimetry, with a focus on achieving deeper insights into the interaction mechanisms of fluoxetine, memantine, and galantamine with irisin. ITC is a multidimensional approach that is routinely used to discover the protein–ligand system’s thermodynamic parameters and molecular forces involved in the binding processes [30]. The formation or dissociation of the protein–ligand complex results in either the release or the absorption of heat in the sample cell, which is measured for a reference cell filled with a corresponding buffer. The deduction of dilution heat corrected the results of the sequential titrations for all three ligands. The binding constant (*K*), the number of binding sites (*n*), entropy change (Δ*S*), and enthalpy change (∆*H*) were determined directly by curve fitting [19]. Figure 5A–C shows the ITC isotherm obtained for irisin–fluoxetine, irisin–memantine, and irisin-galantamine. The upper panel demonstrates each peak in the isotherm representing a single injection of different ligands (fluoxetine, memantine, and galantamine) into the irisin solution. The lower panel depicts the integrated plot of heat released per injection as a function of molar ratio of ligand to the protein. It is clear from these isotherms that fluoxetine, memantine, and galantamine spontaneously bind to irisin, forming a stable complex. Additionally, the negative heat deflection for all three ligands suggests that the binding is an exothermic process. Table 3 shows different thermodynamic parameters obtained for the binding of ligands with irisin. ITC results further validated the fluorescence binding observations, affirming the binding of fluoxetine, memantine, and galantamine with irisin, while fluoxetine showed the maximum affinity for irisin.

### 2.3. Molecular Docking

**Figure 6:**
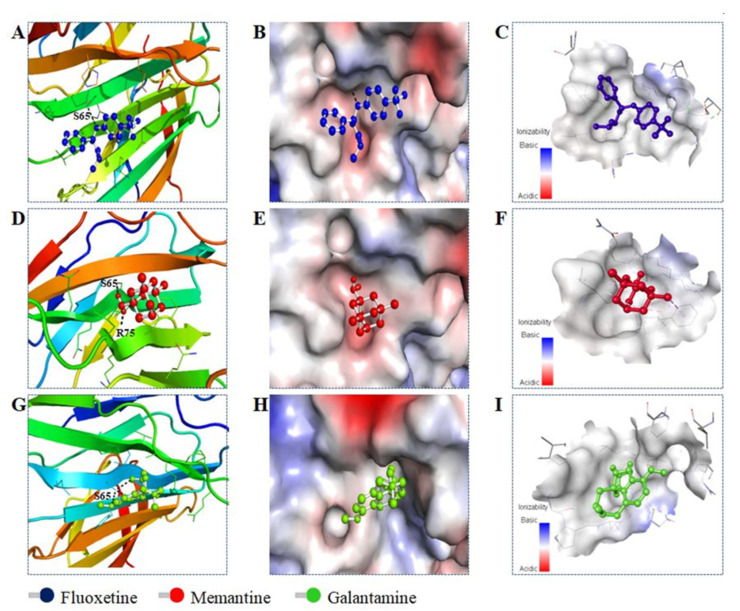
(**A**,**D**,**G**) Cartoon representation showing the interaction of irisin with fluoxetine, memantine, and galantamine, respectively. (**B**,**E**,**H**) Potential surface representation of the binding pocket and its interaction with fluoxetine, memantine and galantamine, respectively. (**C**,**F**,**I**) Zoomed surface view of the irisin binding pocket occupied by fluoxetine, memantine, and galantamine, respectively.

**Figure 7:**
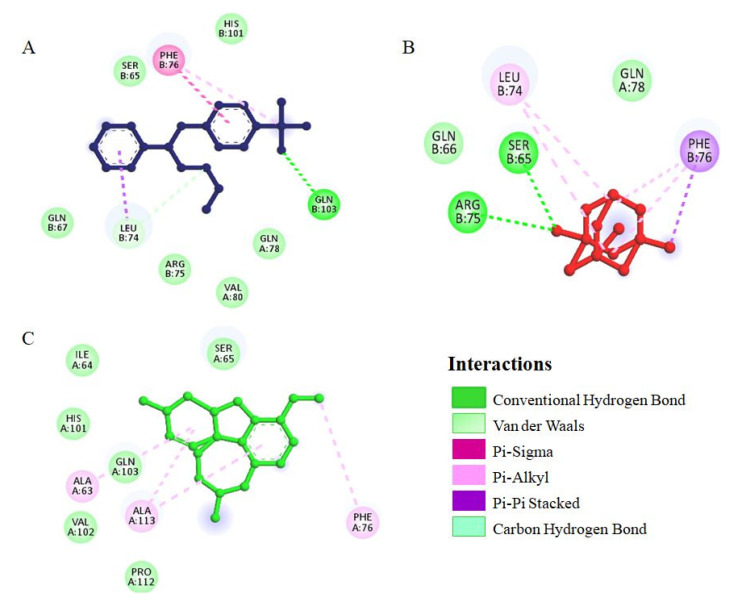
Two-dimensional structural representation of irisin residues interacting with (**A**) Fluoxetine (**B**) Memantine, and (**C**) Galantamine.

Molecular docking is a useful technique for obtaining mechanistic details of protein–ligand interactions at a molecular level, further supporting the identification of interacting residues and calculating affinity scores. This study utilized a blind docking approach to identify all possible interactions between irisin and different drugs. The results of blind docking of each compound, that is, all the potential binding sites determined for each compound have been shown in Figure S1. Additionally, their binding scores also have been provided in Table S2. For the irisin–fluoxetine interaction, the predicted affinity score was estimated as −6.3 kcal/mol, which is significant and affirms that fluoxetine is a possible binding partner of irisin. The docking analyses of memantine and galantamine with irisin also showed significant affinity, and the binding scores were 5.7 kcal/mol and −5.6 kcal/mol, respectively. The docking analysis also supports the fluorescence and ITC results, which revealed that fluoxetine binds to irisin with the highest affinity. Figure 6 depicts the irisin in complex with docked fluoxetine, memantine, and galantamine shown in the ball-and-sticks model. Various interactions were shown by fluoxetine with key irisin residues, including one hydrogen bond with Gln103 and a few other non-covalent interactions. The 2D plot of the irisin–fluoxetine interaction showed a detailed analysis of all important residues of irisin interacting with fluoxetine (Figure 7A). Fluoxetine showed multiple van der Waals interactions with Ser65, Gln67, Arg75, Gln78, Val80, and His101 of irisin.

Figure 7B depicts 2D plot showing all the key residues of the irisin involved in interaction with memantine. Memantine was found to form two conventional hydrogen bonds with Ser65 and Arg75, highlighting the importance of this complex. Moreover, memantine showed van der Waals interaction with Gln78 of irisin. The 2D plot of irisin-galantamine interaction (Figure 7C) showed several van der Waals interactions by galantamine with Ile64, Ser65, His101, Val102, Gln102, and Pro112 of irisin. All possible docked conformers of fluoxetine, memantine, and galantamine were analyzed, and it was observed that Ser65 of irisin is a critical residue involved in the interaction with all three drugs. Both fluoxetine and memantine were found to preferentially bind with similar binding sites of irisin. Therefore, taken altogether, these docking results indicate that fluoxetine more efficiently binds with irisin as compared to memantine and galantamine, also complementing the spectroscopic results.

Therapeutic efficacy of most of these drugs is often limited by their short half-lives due to renal clearance and degradation before reaching the target site [31]. During the administration of drugs through an oral route, it is also necessary to ensure that these compounds are insoluble in the stomach, where there are several enzymes and acids that can result in substantial loss in these drugs [32]. Furthermore, these drugs must be dissolvable and should be adsorbed in the intestine through intestinal mucosa [33]. Hence, for chronic and persistent pathological conditions, there is a requirement of non-toxic, long-term, and sustainable drug delivery systems. There are several polymer materials that have been implicated in the drug delivery process in the form of nanoparticles, microcapsules, micelles, microspheres, gels, and fibers [34]. For drug delivery, interest in using protein-based biopolymers has dramatically increased in recent years due to its advantageous characteristics, including biocompatibility, biodegradability, water solubility, and non-toxicity [32]. It has been observed that natural biological molecules such as protein and polysaccharides have excellent drug carrier system properties. The drugs, applied by any means, are transported by blood and encounter a multitude of proteins along with various cellular components. Therefore, it is predicted that using proteins or hormones in the drug delivery systems in the form of protein–drug nanoparticles, fusion proteins, or pro-drugs can be an effective therapeutic approach [35]. In the same context, an interaction study between irisin (a hormone) and AD drugs has been carried out as a preliminary study for understanding the interaction mechanism implicated in designing irisin–drug conjugates. Fluoxetine is an approved drug for AD-related symptoms and is commonly used for the treatment of anxiety and depression, and therefore it is predicted that the complex of irisin–fluoxetine can be considered as a potential candidate in the drug delivery system. Memantine also has shown significant binding with irisin, and thus is a possible binding partner of irisin. There are several proteins including transferrin and albumin that are being used as a drug carrier, so it is hypothesized that irisin can also serve as a good carrier for the delivery of drugs, as it is also known to have diverse functions in various body parts [17,26]. Moreover, investigations showed that serine residue of irisin is critically involved in interaction with all the three drugs, so it can also be implicated in designing of drug conjugates with irisin.

## 3. Materials and Methods

The irisin expression construct (*pET15b*-His-3C-*irisin*) was procured from Addgene (122612). For the expression of irisin, the C41-DE3 strain of *E. coli* was used. Ampicillin, memantine, galantamine, and fluoxetine were obtained from Sigma-Aldrich Co. (Saint Louis, MO, Missuori, USA). For buffer preparations, analytical -grade chemicals were used.

### 3.1. Expression and Purification of Irisin

Irisin was purified according to previously published reports [26,28]. In brief, the expression construct of irisin was transformed in *E. coli* C41-DE3 cells. Maximum protein expression was obtained at 16° C by 0.5 mM IPTG induction (18–20 h). The culture was harvested, and cells were dissolved in lysis buffer (50 mM Tris–HCl buffer pH 7.5, 300 mM NaCl, 0.5 mM β-mercaptoethanol, 5% (*v*/*v*) glycerol, and 1 mM phenyl methane sulfonyl fluoride, and centrifuged at 9000 rpm for 30 min at 4 °C. A Ni-NTA column was equilibrated and the supernatant was obtained after centrifugation was loaded on the column. The protein was eluted out at 250 mM imidazole concentration, and purity was determined on SDS-PAGE.

### 3.2. Fluorescence Spectroscopic Measurements

The fluorescence-based assay was performed and analyzed according to previously published reports to determine the binding affinity of memantine, fluoxetine and galantamine with recombinant irisin [26,36]. The stock solutions of drugs were diluted using double distilled water. A 4 μM concentration of irisin was used, and fluorescence measurements were taken on a Jasco spectrofluorometer FP 6200 (Jasco, Japan). The protein was excited at 280 nm, and the emission range was 300–440 nm. Both the excitation and the emission slit widths were set at 10 nm. All the spectra were reported after taking inner filter effect into consideration [37].

Fluorescence quenching of irisin was mathematically analyzed using different equations, namely, Stern–Volmer (SV) (Equation (1)) [38,39], and the double logarithmic equation (Equation (2)) [40]. 

### 3.3. ITC Measurements

The binding affinities of fluoxetine, memantine, and galantamine with irisin were investigated using a VP-ITC microcalorimeter (MicroCal, Inc, GE, MicroCal, Northampton, MA, USA) at 25 °C following previously published reports [41]. The sample cell was filled with irisin solution while the three ligands were loaded into the rotator syringe. These samples were degassed before loading, and the degassing was carried out in a thermovac chamber for 30 min. The first injection was a false one of 5 µL followed by 10 µL injections. Spacing was set at 280 s, and the stirring speed was kept at 307 rpm. The curve fitting was performed using different binding site models for different ligands. For irisin–fluoxetine, one site model was followed, while for irisin–memantine and irisin–galantamine, four site models were followed due to the best fitting obtained for the isotherm, as followed in previously published literature [42,43]. The final figure was obtained using the MicroCal Origin 8.0 software to find the association constant (*K*_a_), entropy change (Δ*S*), and enthalpy change (Δ*H*).

### 3.4. Molecular Docking

To have insight into the interaction pattern between irisin and drugs, molecular docking was performed. Bioinformatics tools such as PyMOL [44], MGL Tools [45], AutoDock Vina [46], and Discovery Studio [47] were employed for docking and analysis. The structure of irisin was obtained from the Protein Data Bank (PDB-ID-4LSD) in a 3D state. Similarly, 3D structures of memantine, fluoxetine, and galantamine were taken in SDF format from the PubChem database. The PDB file of the receptor (irisin) and SDF files of ligands (drugs) were converted into PDBQT format to perform docking. The ligand structures and receptor files were used for docking by employing the AutoDock Vina software [46]. Since irisin’s binding sites were not known, blind docking was performed, in which the drugs were facilitated to freely move and search for irisin. The results of the docking were saved in a separate directory, and Discovery Studio Visualizer and PyMOL tools were employed to analyze irisin–drug interactions [44,47].

## 4. Conclusions

This study examines the interaction of irisin with FDA-approved drugs for AD, which can serve as a platform for utilizing irisin as a carrier for the delivery of therapeutic drugs. In the present work, we have reported the molecular interactions of irisin with Alzheimer’s drugs, namely memantine, galantamine, and fluoxetine, employing molecular docking and in vitro binding studies. It has been observed from spectroscopic and molecular docking studies that significant binding occurred between irisin and these drugs, with fluoxetine possessing the highest binding affinity to irisin. Fluorescence quenching revealed that fluoxetine binds to irisin with a high affinity, i.e., *K*= 0.21 × 10^7^M^−1^ at 25 °C. ITC further revealed the associated thermodynamic and binding parameters of all three drugs with irisin, suggesting the binding to be spontaneous and exothermic. Additionally, molecular docking also validated the observations. It was found that irisin binds with all three drugs with significant affinity, and a maximum binding score (−6.3 kcal/mol) was observed for irisin–fluoxetine interaction. This study provides a platform for the use of irisin in a drug delivery system for a favorable therapeutic approach for ameliorating several diseases, owing to the various beneficial roles of irisin in the body.

## Figures and Tables

**Table 1:** Values of Stern–Volmer quenching constants obtained from Stern–Volmer equation.

Irisin–Drug	*K_sv_* (10^4^M^−1^)	*R* ^2^
Irisin–Fluoxetine	2.77	0.97
Irisin–Memantine	2.73	0.98
Irisin–Galantamine	1.77	0.94

**Table 2:** Binding parameters obtained from double logarithmic equation.

Irisin–Drug	*K*	*n*
Irisin–Fluoxetine	0.21 × 10^7^M^−1^	1.43
Irisin–Memantine	9.78 × 10^5^M^−1^	1.32
Irisin–Galantamine	0.14 × 10^3^M^−1^	0.56

**Table 3:** Binding and thermodynamic parameters obtained from ITC.

Irisin–Fluoxetine System		
*K*_a_ (Association Constant), M^−1^	∆*H* (Enthalpy Change), cal/mol	∆*S* (cal/mol/deg)
*K*_a1_ = 6.41 × 10^6^ ± 9.66 × 10^4^	∆*H*_1_ = −1.23 × 10^4^ ± 979.4	∆*S*_1_ = −10.3
**Irisin–Memantine system**		
*K*_a1_ = 8.97 × 10^4^ ± 2.9 × 10^3^	∆*H*_1_ = 5744 ± 2.52 × 10^3^	∆*S*_1_ = 41.9
*K*_a2_ = 1.07 × 10^5^ ± 4.2 × 10^3^	∆*H*_2_ = −1.357 × 10^5^ ± 7.41 × 10^3^	∆*S*_2_ = −432
*K*_a3_ = 9.91 × 10^4^ ± 5.5 × 10^3^	∆*H*_3_ = 1.92 × 10^5^ ± 1.74 × 10^4^	∆*S*_3_ = 669
*K*_a4_ = 1.06 × 10^5^ ± 6.1 × 10^3^	∆*H*_4_ = −1.99 × 10^5^ ± 1.65 × 10^4^	∆*S*_4_ = −645
**Irisin–Galantamine system**		
*K*_a1_ = 1.05 × 10^5^ ± 1.7 × 10^4^	∆*H*_1_ = 6763 ± 6.18 × 10^3^	∆*S*_1_ = 45.7
*K*_a2_ = 1.24 × 10^5^ ± 2.3 × 10^4^	∆*H*_2_ = −2.121 × 10^5^ ± 3.19 × 10^4^	∆*S*_2_ = −688
*K*_a3_ = 6.16 × 10^4^ ± 9.1 × 10^3^	∆*H*_3_ = 1.88 × 10^5^ ± 6.12 × 10^4^	∆*S*_3_ = 655
*K*_a4_ = 5.54 × 10^4^ ± 1.0 × 10^4^	∆*H*_4_ = −3.076 × 10^5^ ± 4.49 × 10^4^	∆*S*_4_ = −1.01 × 10^3^

## Data Availability

All the data have been provided in the manuscript.

## Supplementary Materials

The following supporting information can be downloaded at: https://www.mdpi.com/article/10.3390/ijms23115965/s1.
