# Supplementary material for: Unraveling the Binding Mechanism of Alzheimer’s Drugs with Irisin: Spectroscopic, Calorimetric, and Computational Approaches
Source: Int J Mol Sci. 2022 May 25;23(11):5965. doi: 10.3390/ijms23115965 (PMC9180407; doi:10.3390/ijms23115965)
Supplement: Supplementary file 1 [file ijms-23-05965-s001.zip › ijms-1718873-supplementary.pdf]

Supplementary File

Unraveling the Binding Mechanism of Alzheimer's Drugs with Irisin: Spectroscopic, Calorimetric, and Computational Approaches

Rashid Waseem[1], Anas Shamsi[1], Tanzeel Khan[1], Md. Imtaiyaz Hassan[1], Syed Naqui Kazim[1], Mohammad Shahid[2], and Asimul Islam[1]*

[1]*Centre for Interdisciplinary Research in Basic Sciences, Jamia Millia Islamia, New Delhi.* *Email: rashid.waseem439@gmail.com; anas.shamsi18@gmail.com; aligbcb11@gmail.com; mihassan@jmi.ac.in; snkazim@gmail.com; aislam@jmi.ac.in*

[2]Department of Basic Medical Sciences, College of Medicine, Prince Sattam Bin Abdulaziz University, Al-Kharj 11942, Saudi Arabia. Email: dr.shahid90@yahoo.com

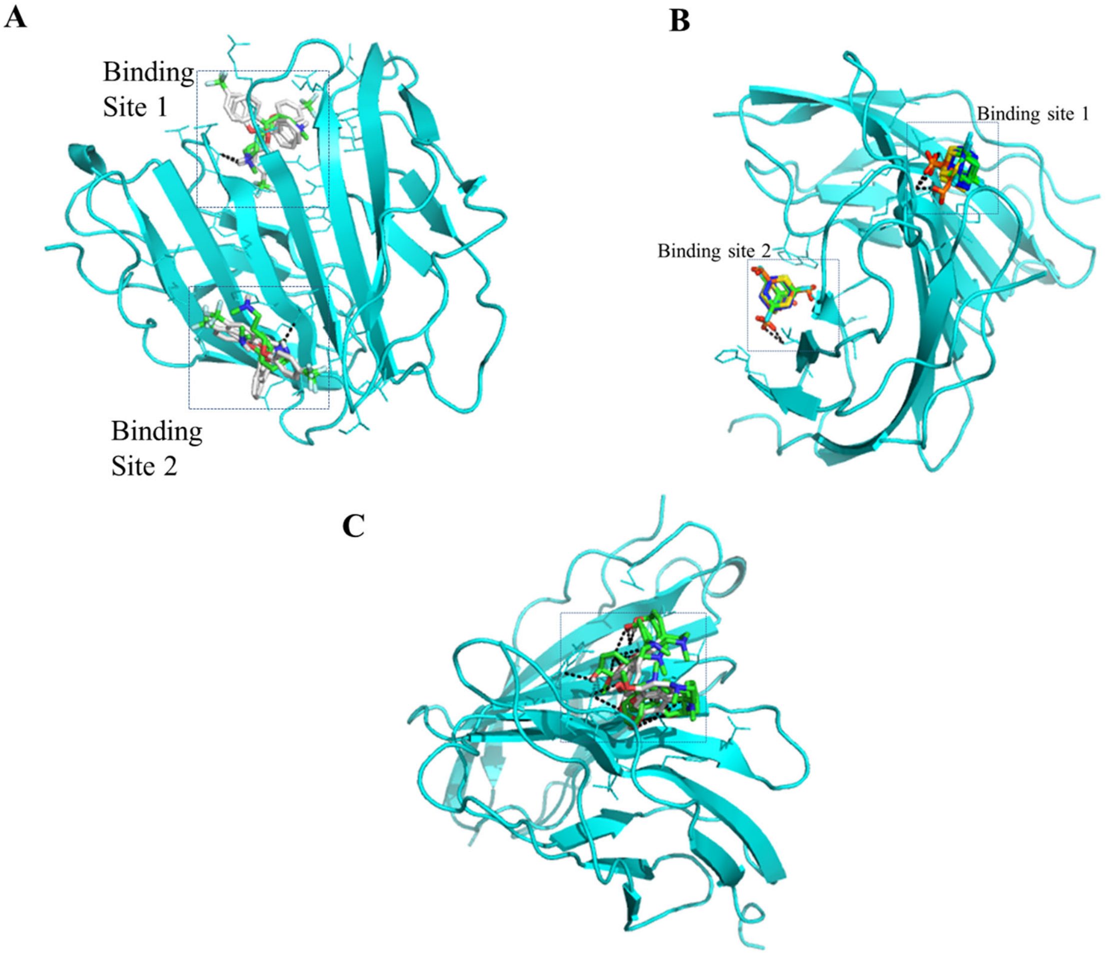

Figure S1: (A) Cartoon representation showing the two different binding sites of irisin for fluoxetine. **(B)** Cartoon representation showing the two different binding sites of irisin for memantine. **(C)** Cartoon representation showing the binding site of irisin for galantamine.

A

Mode	Affinity (kcal/mol)
1	-5.6
2	-5.6
3	-5.6
4	-5.3
5	-5.2
6	-5.1
7	-5.1
8	-5.0
9	-5.0

B

Mode	Affinity (kcal/mol)
1	-5.7
2	-5.4
3	-5.4
4	-5.3
5	-5.3
6	-5.2
7	-4.9
8	-4.9
9	-4.8

C

Mode	Affinity (kcal/mol)
1	-5.6
2	-5.6
3	-5.6
4	-5.3
5	-5.2
6	-5.1
7	-5.1
8	-5.0
9	-5.0

Table S1. Possible binding modes of **(A)** Fluoxetine, **(B)** Memantine and **(C)** Galantamine with irisin and their affinity scores.
